# Use of *Bacillus thuringiensis var israelensis* as a viable option in an Integrated Malaria Vector Control Programme in the Kumasi Metropolis, Ghana

**DOI:** 10.1186/1756-3305-6-116

**Published:** 2013-04-22

**Authors:** Rita Nartey, Ellis Owusu-Dabo, Thomas Kruppa, Sandra Baffour-Awuah, Augustina Annan, Samuel Oppong, Norbert Becker, Kwasi Obiri-Danso

**Affiliations:** 1 Kumasi Center for Collaborative Research in Tropical Medicine, Kwame Nkrumah University of Science and Technology, Kumasi, Ghana; 2 Department of Theoretical and Applied Biology, Kwame Nkrumah University of Science and Technology, Kumasi, Ghana; 3 Bernhard Nocht Institute for Tropical Medicine, Hamburg, Germany; 4 German Mosquito Control Association (KABS), Waldsee, Germany

**Keywords:** Anopheles gambiae, Bacillus thuringiensis var israelensis, Water dispersible granule, Malaria vector, Microbial larvicide, Mosquito control, Kumasi, Ghana

## Abstract

**Background:**

Integrated Vector Control (IVC) remains the approach for managing the malaria-causing vector. The study investigated the contribution of *Bacillus thuringiensis israelensis* (*Bti*) in the control of malaria by targeting the larvae and also mapped and documented major breeding sites in the Kumasi metropolis, Ghana.

**Methods:**

Using a hand held GPS receiver unit, major breeding sites within the metropolis were mapped out during the larval survey. Mosquito larvae were then collected from the breeding sites and reared in an insectary to obtain an F1 generation for laboratory bioassays. The minimum effective dosage of *Bti* Water Dispersible Granular (WDG) formulation was determined by a series of bioassays. Based on the results obtained in the laboratory, the optimum effective dosage of *Bti* formulations against naturally occurring larvae of the indigenous mosquito species was determined through open field trials.

**Results:**

A total of 33 breeding sites were identified and geo-referenced during the larval surveys with the majority of the breeding sites located in the Asokwa sub-metropolis, Kumasi, Ghana. A *Bti* (3,000 International Toxic Unit (ITU)/mg) concentration of 0.026 mg/l resulted in 50% mortality whilst a concentration of 0.136 mg/l resulted in 95% mortality. Results from the open field trials with *Bti* showed that a dosage of 0.2 kg/ha is as effective as 0.4 kg/ha in suppressing late instars and resulting pupae.

**Conclusion:**

This study reveals that *Bti* at a very low dosage of 0.2 kg/ha is highly effective against *Anopheles* larvae and therefore offers viable options for the management of vector mosquitoes. Further research is needed to extend this to the field in order to determine its ability to reduce malaria incidence.

## Background

Extensive use of chemical insecticides against vector mosquitoes, for the control of malaria and other mosquito borne diseases, for about four decades, has caused development of resistance in vector mosquitoes to these insecticides and hazards to the environment [1-5]. In spite of the sustained and prolonged use of chemical insecticides, these diseases are not only still prevalent but also cause epidemics [2]. Research undertaken to reduce malaria incidence in Ghana and Kumasi in particular, have, over the years focused mainly on the adult vectors. Therefore, to minimize the dependency on chemical insecticides, there is an urgent need to explore alternative measures for the control of vector mosquitoes.

The advantages of *Bacillus thuringiensis var israelensis (Bti)* in comparison to chemical control is its effectiveness at relatively low doses, safety to humans and non-target wildlife, low cost of production in some cases and lower risk of resistance development [6]. Furthermore, the larvae are concentrated in predictable sites that can be easily accessed, treated or manipulated with no chance of larvae escaping [6]. It is the first study to be carried out on *Bti* in Kumasi. It focuses on the contribution of microbial agents in Integrated Vector Control Programmes with emphasis on *Bti* and exploits the application of Geographical Information Systems (GIS) Technology in mapping breeding areas. The results of this work should serve as a reference on malaria risk areas in Kumasi and a guideline for future research on the contribution of microbial agents and the importance of its inclusion in the implementation of future malaria control programmes.

## Methods

### Study area

Laboratory and standardized field trials were carried out at the Kumasi Centre for Collaborative Research (KCCR) on the Kwame Nkrumah University of Science & Technology (KNUST) campus. KNUST is situated about 8 km from the main Kumasi township. It covers an area of about 18 km^2^ of undulating land and lies between latitude 6^o^39’ & 6^o^47’ N and longitude 1^o^26’ & 1^o^40’ W [7].

Data on daily minimum and maximum temperatures and rainfall were available from the meteorological station near the Faculty of Agriculture about 1.3 km from KCCR on the KNUST campus.

### Mosquitoes

Laboratory assays were carried out with third instar larvae of insectary-reared *Anopheles gambiae* larvae which were originally colonized from *Anopheles* larvae collected from a lettuce farm on KNUST campus, about 3 km from KCCR in August 2010, and maintained at the KCCR insectary.

All mosquito larvae used in the laboratory experiments were reared at a room temperature of 28°C, 80% relative humidity and approximately a 12 hour light: 12 hour dark cycle. Larvae were reared in 26 × 24 × 5 cm transparent, rectangular plastic containers filled with 300 ml tap water that had been left in the insectary for at least 48 hours to equilibrate. Larvae were fed by adding a pinch of crushed Tetramin^@^ (Tetra, Germany) fish food spread evenly on the water surface twice daily.

### Bacillus formulations

WDG of the commercial strains of *Bti* (VectoBac^@^ strain AM65-52; Lot number 187-600-PG, 3000 ITU/mg; ValentBioScience Corporation, Illinois, USA) were tested in the laboratory and under field conditions. This was carried out in a similar manner to that described in the WHO Guidelines for Laboratory and field testing of mosquito larvicides [8]. *Bti* WDG formulations were applied as liquid with 250 ml handheld sprayers. It was observed that *Bti* WDG formulation dispersed readily when mixed with water and remained like that for at least several minutes.

### Bioassays

Laboratory assays were conducted with the WDG formulation of VectoBac^@^ to determine the minimum effective doses following the standard testing procedures for microbial test [9]. Twenty-five third instar larvae were randomly collected for the experiment from several containers to compensate for size difference and feeding history which are known to be influenced by larval density [10]. They were then transferred to new disposable 300 ml plastic containers filled with 100 ml of distilled water.

On every test date, fresh stock solutions were prepared and test aliquots made with distilled water. After range finding tests (10), six different test concentrations, 0.001, 0.002, 0.004, 0.008, 0.016 and 0.032 mg/l were chosen. The bioassays were run in the six different concentrations plus controls and replicates.

Each experiment contained an untreated control. The experiments were run in four replicates at the same time and the entire experiments were carried out on three different occasions. Larvae were not fed during the experiments and all tests were run at ambient temperatures ranging between 21°C and 34°C. Larvae were counted and mortality scored after 24 hours. Moribund larvae were considered dead and included in the analyses. Where mortality exceeded 10% in the controls, the experiments were discarded and repeated.

### Open field trials

An open sunlit area of about 28 m^2^ was cleared from all vegetation. Artificial ponds were then created following the experimental design of Fillinger *et al*., [11]. Eighteen 0.6 m diameter plastic bowls were buried at a depth of about 0.2 m-0.3 m in two lines of nine containers. Distances between the containers were 1.5 m. Top soil and mud from the *Anopheles* breeding site at the cabbage farm was added to each of the plastic bowls (one-third of its volume) to provide the abiotic and biotic conditions suitable for mosquitoes. The containers were filled with tap water and maintained at a depth of 0.2-0.3 m. The habitats were then left open for mosquito oviposition. The trials were conducted with offspring of wild *Anopheles gambiae* females that oviposited in the experimental container. Colonization of experimental containers occurred within 4 days and sometimes included larvae of *Culex*.

Experiments were implemented nine days after the containers were set-up to allow third and fourth instar larvae to develop. Water temperatures during the experiments ranged between 23°C and 40°C. After colonization of the containers, completions of the larval life cycle were found to take only 10 days, due to high water temperatures. In order to prevent the emergence of the malaria vector, all habitats were carefully screened for pupae twice daily (visually and with a dipper) and any mature pupae present were removed.

Out of the 18 artificial containers, six served as controls, and each half of the remaining 12 containers were treated with a given concentration of the test formulation. Containers were matched on the basis of larval density so that control and test treatment containers had similar densities at the start of the experiment. *Bti* concentrations were selected on the basis of laboratory results and studies reported elsewhere [12-15].

Treatment concentrations were calculated on the basis of a standard water depth of 0.1 m and a fixed surface area [16,17] irrespective of the actual water depth and this was done to simulate operational procedures. Laboratory tests were conducted under standardized conditions without major abiotic and biotic influences, therefore the LC values represented minimum dosages only. Under field conditions, application rates normally have to be increased up to several times the LC_95_ to obtain sufficient larval control [18]. *Bti* WDG is not considered to show a long residual effect from findings made elsewhere [19] and others; therefore the optimum effective dosage used was the minimum dosage for 100% larval mortality 48 h after application. The microbial larvicides, *Bti* WDG as tested in this setup were at a concentration of 0.2 mg/l and 0.4 mg/l.

The liquid formulations of *Bti* were sprayed evenly over the entire water surface using a 250 ml handheld sprayer. Afterwards, all the containers were examined daily and the average number of larvae and pupae per dip (350 ml capacity dipper, Clarke Mosquito Control products, Illinois, USA) was determined by taking five dips from four different directions of each pond close to the edge and one from the middle. Mosquito larvae were classified as anophelines or culicines and recorded as early (1^st^ and 2^nd^) or late (3^rd^ and 4^th^) instars. All larvae were counted, classified to genus and development stage and then returned to their respective sites and all pupae were removed.

### Data analyses

From the bioassay results, LC_50_ and LC_95_ values were determined using log-probit regression analysis in SPSS software version 16.0 [20]. LC_50_ represents the probability of success or the chance of 50% of the larvae dying and LC_95_, the chance of 95% dying.

The percentage reduction in larval mosquito densities were calculated using the formula of Mulla *et al*.; [21] which takes into account that natural changes (for instance through predation) in the mosquito larval populations are taking place at the same level and rate in both treated and untreated sites:

$$\mathrm{Percentage}\;\mathrm{reduction}=100-\left(C1/T1\;x\;T2/C2\right)\;x\;100$$

Where C1 and C2 describe the average number of larvae in the control containers for pre- and post-treatment. While T1 and T2 represent those for the containers treated with experimental formulations. Average number of larvae and pupae per dip in control and treatment sites in field tests were compared using Two-way ANOVA. The tests were implemented separately for each sampling day comparing average numbers of immature stages in the controls with treatments. All analyses were carried out using version 9.2 of SAS statistical software package [22].

## Results

### Mapping of breeding sites

**Figure 1:**
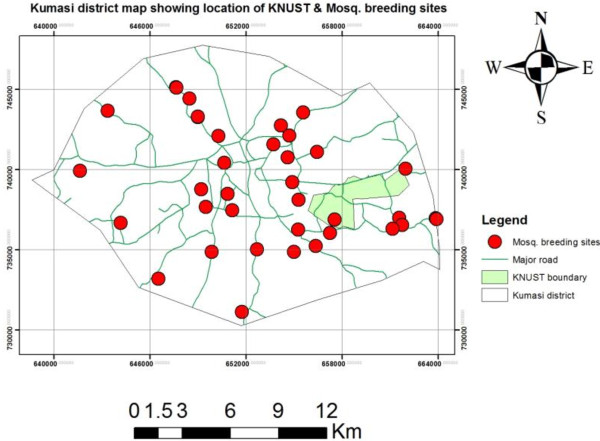
Map of Kumasi district showing location of boundary map of KNUST and breeding site.

In all, 33 major mosquito breeding sites within the Kumasi metropolis were mapped out (Figure 1). Of the five Ghana Health Service sub metropolitan divisions in the Kumasi metropolis, the Asokwa sub-metro had the highest number of breeding sites (9), followed by the Bantama sub-metro (7) whilst the Subin sub-metro recorded the least (5) (Table 1).

**Table 1:** Characteristic of major mosquito breeding sites in the five sub-metropolitan areas in the Kumasi

**Sub-metropolis**	**Number of breeding sites (%)**	**Characteristicsof breeding sites**
ASOKWA	9	Vegetable farming, uncovered choked gutters and drains.
BANTAMA	7	Edges of streams, drainage channels, sugar cane and vegetable farming and temporary pools created after rains.
MANHYIA NORTH	6	Uncovered choked gutters and drains, water collected in foundations of residential buildings and market stalls under construction
MANHYIA SOUTH	6	Edges of streams, abandoned sand winning sites.
SUBIN	5	Edges of streams, cattle hoof prints and open drains

Generally, the mosquito breeding sites that were identified within the study area included sand pools or ponds, edges of streams, drainage channels on sugar cane and vegetable cultivation sites and temporary pools created after rains, choked drains, pools of water collecting in foundations of uncompleted buildings and abandoned sand winning sites (Table 1).

Out of a total of 33 breeding sites identified during the surveys, three were temporary breeding sites and 30 were permanent breeding sites. The breeding sites that had clear and shallow water produced higher densities of *anopheline* larvae, while domestic waste water in choked gutters contained high numbers of *Culex sp.*

### Laboratory assays

**Figure 2:**
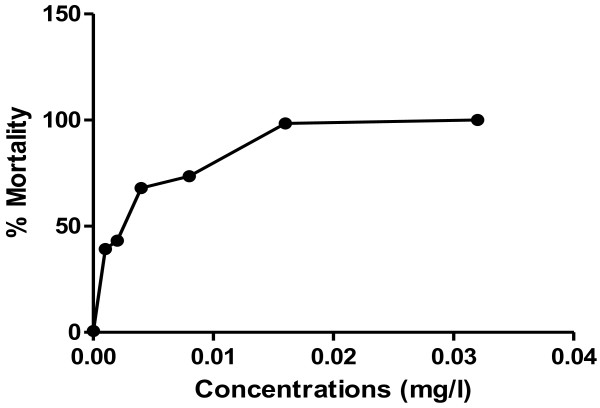
**The graph showing the percentage mortality of *Anopheles *larvae at various concentrations after 24 hours exposure to the *Bti *formulation.**

Larval mortality was determined by counting the live third instar larvae of *Anopheles gambiae* remaining after 24 hrs exposure to *Bti* WDG (VectorBac^@^, 3000 ITU/mg). Moribund larvae were counted and added to dead larvae in calculating the percentage mortality. The *Bti* concentration of 0.026 mg/l resulted in 50% mortality while the 0.136 mg/l concentration caused 95% mortality (Figure 2).

### Open field trials

Anopheline and Culicine mosquito larvae were detected 4 to 5 days after the artificial habitat was set-up. About 13% of the total larval population were *Culex* and since there was no significant difference in terms of the impact of the *Bti*; on *Anophelines* and *Culicines* larvae in the standardised field trials, results from both genera were pooled for all analyses. The early instars represented the first and second instar larvae whilst the third and fourth instar larvae were the late instar larvae. In both the rainy and dry season, *Bti* WDG resulted in 100% mortality within 24 hours at all doses of application.

The *Bti* was effective against both the early and late instars of *Anopheles gambiae* species 24 hrs post treatment, yielding more than 95% mortality. The efficacy of the *Bti* was readily demonstrated by a high initial mortality in larval population of the late instars.

#### Dry season

**Figure 3:**
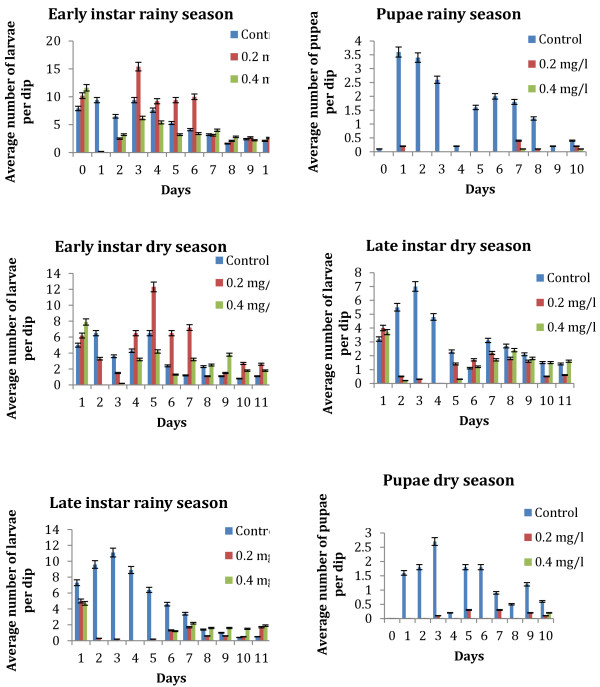
**Effect of Bti WDG formulations on densities of late and early instar *Anopheles gambiae *in simulated field trials with varying conc (s) for dry season ('A' is for Dry season, 'B' is Rainy season).**

The initial (Day 0) average number of *Anopheles* larvae per dip before the *Bti* formulation was added (pre-treatment) were 8.2 for the control and 10.2 and 11.6 for the treatment concentrations (T1 and T2 respectively) (Figure 3).

Considering the late instars only, reduction rates of 51-100% could be observed up to the fourth day after treatment. *Bti* was very effective against the late instars reducing the population by 93-97% within 24 hour post treatment for T1 and T2 concentrations respectively. The reduction even increased gradually to 100% within 72 hours. Generally, larvicidal impact on the late instars remained high up to day 4 post treatment with *Bti* (Figure 3).

*Bti* was effective against early instars of *Anopheles gambiae* with a reduction of 96%-100% of the larval population within 24 hours. This effect lasted up to day 2 after application. The larvicidal impact decreased on the 3^rd^ day, where percentage mortality was recorded at 0% and 53% for T1 and T2 respectively.

#### Rainy season

In the rainy season, the average number of *Anopheles* larvae per dip recorded before the *Bti* formulation was added (pre-treatment) was slightly higher than the numbers recorded in the dry season (Figure 3).

Considering the late instars only, reduction rates of 59-100% could be observed up to the fifth day after treatment. *Bti* was very effective against the late instars reducing the population by 95-100% within 24 hour post treatment. Larvicidal impact on the late instars in the rainy season remained high up to day 4 post treatment with *Bti*.

*Bti* was effective against early instars of *Anopheles gambiae* with a reduction of 98-100% of the larval population within 24 hours. This effect lasted up to day 2 post application. The larvicidal impact decreased on the 3^rd^ day, where reduction in larval density was recorded as 0% and 55% for T1 and T2 respectively (Figure 3).

Though the *Bti* formulation resulted in high mortalities on the early and late instars of *Anopheles gambiae* with over 97% reduction within 24 hrs, it showed drastic reduction 48 hrs after application, as dips taken three to four days after treatment indicated quick and continuing re-colonisation of all treated sites by early instars. Both concentrations tested (0.2 mg/l and 0.4 mg/l), were equally effective up to 3 days post-treatment for the total number of larvae and up to 4 days when considering the late instars only. There were no statistically significant differences between the 0.2 mg/l and 0.4 mg/l treatment concentrations.

### Pupation levels

Pupation, which determines to a large extent the number of adult mosquitoes that will emerge , was very low in all the treatment ponds (Figure 3). All treatments were effective at lowering pupal populations and an overall reduction in mosquito emergence was achieved.

*Anopheles* adults that emerged from pupae collected from the control containers were identified during the study period, using the morphological keys of Gillies and Demeillon [23] and Gillies and Coetzee [24], and found to be *Anopheles gambiae* Giles complex.

### Climate

**Figure 4:**
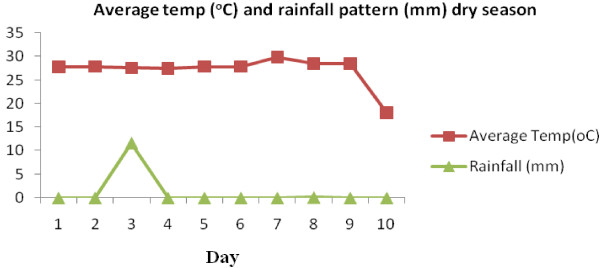
Average temp (°C) and rainfall pattern (mm) dry season.

**Figure 5:**
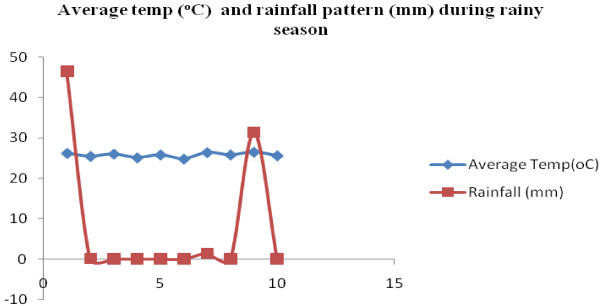
Average temp (°C) and rainfall pattern (mm) during rainy season.

The average minimum and maximum temperature and the daily rainfall patterns during the study period from 7^th^ July 2011 to 17^th^ November 2011 are represented in Figures 2 and 3. The field trials for the dry season, which were conducted from the 8th to 17th November 2011, and for the rainy season 7^th^ July to 16^th^ July 2011 are shown in Figures 4 and 5 respectively.

For the first 9 days of the experiment carried out in the dry season, temperatures remained constant (27.25°C) until day 10, when it dropped to 25.85°C. On the third day, rain showers (11.6 mm) were recorded and lasted for five hours but did not have any impact on the average temperature (Figure 4). Rainfall was recorded twice during the experimental period on day 1 (46.5 mm) and day 9 (18.1 mm) of the rainy season test, but did not seem to have any impact on the results. Temperatures however remained constant (Figure 5).

## Discussion

Our findings indicate that laboratory bioassays, concentrations of 0.026 mg/l and 0.136 mg/l resulted in 50% and 95% mortalities respectively after 24 hours of exposure to *Bti* WDG (VectorBac^@^, 3000 ITU/mg). This is similar to 0.039 mg/1 (LC_50_) and 0.132 mg/1 (LC_95_) in Gambia [20] and 0.021 mg/1 (LC_50_) and 0.21 mg/1 (LC_95_) in Kenya [11]. The similarities in vulnerability of the *Anopheles* larvae to the *Bti* in studies conducted in East Africa [11,25] and West Africa [20] confirms that biolarvicidal activity is intrinsic to the mosquito but not ecologically determined [26]. However, the concentration, 0.136 mg/1 obtained in this study as the LC_95_ depicts that *Bti* formulation is highly effective against *Anopheles* larvae in the Kumasi metropolis.

Concentrations of 0.2 kg/ha and 0.4 kg/ha that were subsequently tested in open field trials revealed that both concentrations tested were equally effective up to 3 days post-treatment for the total number of larvae and up to 4 days when considering the late instars only. Early instars were found to be more susceptible to the *Bti* formulation as compared to the late instars.

The controlled field experiments during both the rainy season and the dry season had the residual effect for *Bti* to be 3 to 4 days and compares to studies carried out in several areas; in Cuba [27], Thailand [28], Kenya [11] and Gambia [20].

The bowls were exposed to the whole array of environmental factors such as water quality, sunlight, which are also typical for other mosquito breeding places and the high numbers of immature stages of *Anopheles gambiae* indicate that water bodies on KNUST campus represent dominant sources of *Anopheles gambiae*. The results of the control bowls demonstrate that there is a steady supply of young instars from eggs, which were not affected by the larvicide. The observed fluctuations in larval populations have also been reported in other studies [28]. *Bti*, however, generally showed a higher activity against late instars than early larval stages (Figure 3). The consequences are that interventions have to be focused on late instar larvae. This is especially important when breeding sites are retreated.

The number of larvae that were oviposited in the bowls during the controlled field trial in the rainy season were more in number than the number of larvae that oviposited during the dry season and this may be due to the fact that *Anopheles* breeds mostly during the rainy season than in the dry season [29] and there are reports of relatively high incidence of malaria in Ghana during the rainy season than in the dry season [30].

*Anopheles* adults that emerged from pupae collected from the control containers during the study period and identified using the morphological keys of Gillies and Demeillon [23] and Gillies and Coetzee [24], were found to be *A. gambiae* Giles complex. The reason could be that abiotic conditions were conducive for *Anopheles gambiae* species.

The results of this study show that the younger-instar larvae were more susceptible to the *Bti* formulation than older instars. This was observed in both the rainy season and the dry season (Figure 3). The reason could be that late fourth instars have ceased feeding or feed little before pupation and are much less susceptible because of lack of ingestion of a lethal dose in a short period of time. This is because pre-pupae and pupae are refractory to *Bti* because they do not feed and therefore do not ingest the toxic particles [31].

Secondly, early instars will definitely be killed by dosages and concentrations that will induce some mortality in older larvae [31]. In asynchronous species such as *Culex*, *Anopheles* and some *Aedes*, all larval instars prevail in the breeding environments and since it was observed in this study that early instars were more susceptible to the *Bti* formulation, it will be prudent to administer the formulation more often in order to kill the early instars before they mature, and also, in administering *Bti*, concentrations should be targeted at killing late instars. Thirdly, the administration of maximum dosages geared to kill older larvae will be necessary to control these heterogeneous larval populations [31].

It was also observed that the containers with high larval density recorded lower instar mortality compared to containers with low larval density after the *Bti* formulation was administered. This observation confirms findings made by Becker *et al.*[32] and Nayar *et al.*[33]. Mulla *et al.*[31] also showed that denser populations of larvae (50–100 larvae per dip) will require 1.5-2 times more material than the low-density populations (5–20 larvae per dip) to yield equal mortalities. Therefore, it will be necessary in the field to make subjective adjustments of rates of *Bti* applications depending on prevailing larval densities [33].

Additionally, containers that had high rates of organic pollution and the presence of colloidal particles had lower larval mortality compared to the other containers that had less colloidal particles. This could be due to the fact that in the presence of organic and floating materials, fewer toxin particles are ingested by the larvae. Moreover, the availability of crystals will be decreased by their adsorption onto suspended particles followed by a slow sedimentation. In both cases (high density and pollution), higher rates of application will be necessary to control mosquito larvae [31,32]. Ohana *et al*., [34] have also shown that contact with mud of a sporal culture of a mutant resulted in an immediate disappearance of the larvicidal activity but had no influence on viability. Thus inactivation of the toxic activity of *B. thuringiensis* subsp. *israelensis* in the mud was a reversible process and was due to bacteria adsorption on the soil particle thus making the bacteria and its toxins inaccessible to the larvae. Similarly, Margalit and Bobroglo [35] reported that the efficacy of *Bti* against 2^nd^ stage larvae of mosquitoes decreased when organic matter was present in the water, or when sterilized silt was added.

During this study, the dominant predator was the tadpole and this may be because the trials were conducted on a wetland. It was however observed that in experimental tubs or containers where their numbers increased the number of early and late instars decreased or were relatively lower. The predation-prey association could not be specifically determined just by counting larvae alive at any point to the number of tadpoles present because the larvae might have died due to other external influence other than predation. In nature, *Anopheles gambiae* typically breed in temporal habitat where predators are not present or their presence is relatively low [36].

The number of late instar observed after *Bti* treatment during the rainy season was more than the number noted during the dry season. This may be due to the temperature of the water during the rainy season.

Nayar *et al*. [33] showed that lower water temperatures (15°C) slows the development of larvae, with the result that larvae consume fewer nutrients (and also less endotoxins) and apparently become less susceptible. Higher temperatures (35°C) accelerate development of larvae, with the result that the larvae consume more nutrients and become more susceptible. Although *Bti* was found to be active at low temperatures, its effectiveness may be reduced in cold water due to a cessation or a low rate of feeding of some species of larvae, larval diapause and a decrease in metabolic rate [37]. Similar observations were reported on other mosquito species by Mulla *et al*. [31] and Becker *et al*. [32]. Thus, higher field applications rates of *Bti* may be necessary during the rainy season than during the dry season in order to achieve the same level of control.

Finally, increased sunlight has been shown to lower the efficacy of *Bti*[32]. Therefore, in Ghana where the intensity of sunlight as well as the water temperatures is high especially in the dry season, the combined effect of higher intensity of sunlight and high temperatures can reduce the potency of *Bti* formulations substantially. This suggests that ideally, field applications of *Bti*, should be made during the later part of the day (after 5:00 pm) rather than in the morning hours, particularly during the dry season.

In both the rainy and dry season, there was a re-emergence of larvae after the 4^th^ and 5^th^ day post application of the *Bti* (Figure 3). This suggests that *Bti* has very low residual effect and the observation made in this study conforms to findings made by Karch [38] and others in the Democratic Republic of Congo, who found that larval population begun to recover 5–7 days after treatments at the latest, irrespective of the *Bti* concentration applied (2000–5000 ITU/l, in 0.1 m water depth). This lack of residual effects of *Bti* has been reported previously by Das and Amalrg [39] in Western Kenya.

In the study conducted in the Democratic Republic of Congo, Karch [38] proposed a surface application regime of once every week for *Bti* to achieve >95% reduction on mosquito emergence from breeding sites. Results from the open field also showed that a very low dosage of 0.2 kg/ha is required to effectively suppress late instars and the resulting pupae as seen in Figure 3.

This value corresponds well with the LC_95_ of the laboratory tests and represents the optimum effective dosage to control *Anopheles gambiae* in Ghana. This low application dosage offers the possibility of keeping operational cost low even if weekly treatments, caused by the absence of residual activity, have to be considered.

The results point to the fact that, with commercially available microbial, weekly larviciding using *Bti* will be necessary in Ghana (especially during the rainy season) since the cost of *Bti* is relatively low and the development of resistance is unlikely. From this research, very low dosages of 0.2 kg/ha and 0.4 kg/ha lead to the optimal suppression of the mosquito larvae and pupae and this is consistent with results from East Africa [25;17].

Nevertheless, it would be useful to explore whether greater persistence could be achieved with alternative products. Organophosphates like temephos, appear to be less useful since they rarely show much persistence compared with microbials. Moreover, organophosphates can have a negative impact on non-target organisms and need careful resistance management. Microbial larvicides have several advantages over other mosquito control agents. This includes environmental safety and safety for human consumption; for instance when applied in drinking water [9], in addition to its high efficacy thus makes them powerful vector control agents in Africa and other parts of the tropics.

The results show that the major malaria vectors in Ghana are highly susceptible to *Bti* under laboratory and field conditions. Although in this study, we created as much as possible the natural habitat of the mosquito, we are unable to say that these findings when replicated within the natural environment in large-scale trials would result in the same outcome. We therefore recommend community open field interventions to both determine the cost effectiveness of the intervention for policy action and also to be able to actually translate the findings into actual reduction in malaria incidence within the Kumasi Metropolis.

## Conclusion

The minimum effective dosages of *Bti* formulation with 3,000 International Toxic Units (ITU)/mg were 0.026 mg/l for LC_50_ and 0.136 mg/l for LC_95_.

This study confirms the lack of residual activity of *Bti* and therefore recommend that it be applied weekly especially during the rainy season. The cost of weekly application in consideration of reduction in transmission intensity should therefore be carefully assessed.

Results from the open field trials with *Bti* however showed that a very low dosage of 0.2 mg/l is required to effectively suppress late instars and resulting pupae, and such low application dosages offer the possibility to keep operational costs low even if weekly treatments due to the lack of residual activity of *Bti* have to be considered

The target mosquito larvae tested (larvae of *Anopheles gambiae* complex) were extremely sensitive to the *Bti* formulation, with the most sensitive stage being the early instars.

## Abbreviations

Bti: Bacillus thuringiensis var israelensis

## Competing interest

The authors declare that they have no competing interests.

## Authors' contribution

RN data collection and field work, EOD conceptualization analysis and manuscript drafting, TK field work and proof reading, SBA data collection and coordination, AA manuscript drafting, SO field work and manuscript drafting, NB conceptualization analysis, KOD critical revision of manuscript for intellectual content. All authors read and approved the final version of the manuscript.
